# Association of Law Enforcement Seizures of Heroin, Fentanyl, and Carfentanil With Opioid Overdose Deaths in Ohio, 2014-2017

**DOI:** 10.1001/jamanetworkopen.2019.14666

**Published:** 2019-11-08

**Authors:** Jon E. Zibbell, Arnie P. Aldridge, Dennis Cauchon, Jolene DeFiore-Hyrmer, Kevin P. Conway

**Affiliations:** 1 RTI International, Atlanta, Georgia; 2 Harm Reduction Ohio, Granville; 3 Ohio Department of Health, Columbus

## Abstract

This cross-sectional study examines the association of law enforcement seizures of heroin, fentanyl, and carfentanil with opioid overdose deaths in Ohio from 2014 to 2017.

## Introduction

The United States continues to experience an unprecedented overdose crisis. Fentanyl overdose deaths increased 525% from 3105 in 2013 to 19 413 in 2016 and then increased 45.2% from 19 413 in 2016 to 28 466 in 2017, with fentanyl overdose deaths outpacing deaths from both prescription opioids and heroin in 2016 and 2017.^1,2^ The Drug Enforcement Administration^3^ reported a 5-fold increase in national law enforcement seizures of illicitly manufactured fentanyls, and fentanyl overdose deaths increased substantially over this period. The Centers for Disease Control and Prevention^4,5^ has endorsed using law enforcement drug seizures as a proxy indicator for the illicit drug supply, and a joint investigation between the Centers for Disease Control and Prevention and the state of Ohio^6^ demonstrated concurrent increases in fentanyl overdose deaths and fentanyl seizures. Less known, however, is whether there are associations between law enforcement drug seizures and drug overdose deaths.

## Methods

Ohio’s Bureau of Criminal Investigation provided drug seizure information associated with state and local drug cases from the state’s 88 counties that were tested by 3 public laboratories from 2014 to 2017.^6^ Each record contained a list of drugs detected by qualitative testing via gas chromatography–mass spectrometry, the confiscation date and county, and the weight in grams. We first constructed monthly statewide counts for seizures that contained heroin, fentanyl, carfentanil, cocaine, and methamphetamine and then calculated the percentage of seizures of heroin, cocaine without heroin, and methamphetamine without heroin that also contained fentanyl or carfentanil. Overdose death data were obtained from the Ohio Department of Health from January 2014 to December 2017 and were rereviewed and recoded for fentanyl or fentanyl analogue detection. Overdose deaths were those for which the cause of death, according to *International Statistical Classification of Diseases and Related Health Problems, Tenth Revision* codes, was unintentional (X40-X44), suicide (X60-X64), homicide (X85), or undetermined intent (Y10-Y14). We coded overdose deaths as opioid-involved if *International Statistical Classification of Diseases and Related Health Problems, Tenth Revision* codes T.40.0, T.40.1, T40.2, T40.3, T40.4, or T40.6 were indicated, or if the Ohio Department of Health’s rereview included a separate flag for fentanyl or fentanyl analogues. We summed opioid-involved deaths to yield a monthly, statewide count.

Drug seizures are presented for heroin (29 917 seizures), cocaine (24 462 seizures), and methamphetamine (20 957 seizures) by year from 2014 to 2017, including the percentage of fentanyl or carfentanil in each drug category. Percentages of fentanyl or carfentanil are presented for a subsample with nonmissing weights by 3 weight strata (<1 g, >1 g but ≤30 g, and >30 g), with mean, median, and 99th percentile weights provided for seizures greater than 30 g. A Mantel-Haenszel test compared the distribution of fentanyl or carfentanil across weight categories over the 4-year period. A multivariate, generalized, autoregressive, conditional-heteroskedasticity model estimated the association between drug seizures and drug overdose deaths. This cross-sectional study received a full review and was approved by the institutional review board at the Ohio Department of Health. All data were administrative records and the study did not require an informed consent process. All analyses were completed using Stata MP statistical software version 15.1 (StataCorp). Additional model details are included in the eAppendix in the Supplement. All statistical tests were 2-sided, and *P* < .05 was considered statistically significant.

## Results

Of the 15 104 overdose decedents from 2014 to 2017, 34.3% were female and 1.8% were Hispanic (race was not reported), with a median age of 40 years (interquartile range, 31-51 years). The mean (SD) monthly number of opioid-involved overdose deaths was 260 (78). Of the 29 917 seizures of heroin identified, 23 175 (77.5%) included weights in grams. The percentage of these containing fentanyl or carfentanil increased from 3.4% in 2014 to 48.6% in 2017. Most of the increase involved seizures weighing less than 30 g. By 2017, 52.0% of heroin seizures less than 1 g and 11.2% of heroin seizures greater than 30 g contained fentanyl or carfentanil. Overall, changes in the distribution of the percentage of heroin seizures containing fentanyl or carfentanil over time and across weight categories were statistically significant (χ^2^ = 3528; *P* < .001). Among 18 276 cocaine seizures with weights available in 2017 not containing heroin, 7.1% contained fentanyl or carfentanil, all of which were less than 1 g. Among 558 cocaine and 753 methamphetamine seizures weighing more than 30 g, less than 0.5% contained fentanyl or carfentanil (Table).

**Table.** Percentage of Law Enforcement Seizures of Heroin, Cocaine, and Methamphetamine With Fentanyl or Carfentanil by Seizure Weight, Ohio, 2014-2017^a^

Drug	Seizures, No. (% With Fentanyl or Carfentanil)			
	2014	2015	2016	2017
Heroin				
All seizures	7715 (3.4)	9151 (9.0)	7809 (25.0)	5242 (48.6)
All seizures with weight	5869 (3.3)	6778 (9.0)	6028 (25.4)	4500 (49.8)
By seizure weight, g^b^				
≤1	4654 (3.5)	5236 (9.9)	4767 (27.5)	3576 (52.0)
>1 but ≤30	1132 (2.4)	1412 (6.0)	1141 (18.0)	835 (44.7)
>30^c^	83 (3.6)	130 (3.9)	120 (9.2)	89 (11.2)
Cocaine, with no heroin present				
All seizures	4898 (0.7)	5599 (1.7)	7102 (4.9)	6863 (11.3)
All seizures with weight	3727 (0.3)	3955 (0.4)	5197 (2.1)	5397 (5.9)
By seizure weight, g^b^				
≤1	2568 (0.5)	2545 (0.6)	3419 (2.7)	3611 (7.1)
>1 but ≤30	1071 (0.0)	1274 (0.0)	1607 (0.9)	1623 (3.8)
>30^d^	88 (0.0)	136 (0.0)	171 (0.6)	163 (0.0)
Methamphetamine, with no heroin present				
All seizures	2517 (0.2)	3576 (0.5)	5519 (1.6)	9345 (2.9)
All seizures with weight	1610 (0.1)	2292 (0.0)	4127 (0.4)	7764 (1.2)
By seizure weight, g^b^				
≤1	1070 (0.1)	1534 (0.0)	2901 (0.5)	5364 (1.5)
> 1 but ≤30	393 (0.0)	539 (0.0)	1031 (0.1)	2208 (0.8)
>30^e^	147 (0.0)	219 (0.0)	195 (0.0)	192 (0.0)

^a^ Seizures are reported from the Ohio Bureau of Criminal Investigation’s 3 state laboratories. Test of statistical significance of percentage of synthetic drugs by seizure weight, by year is the Mantel-Haenszel χ^2^ test with 1 *df*.

^b^ *P* < .001.

^c^ Mean, 192 g; median, 68 g; 99th percentile, 1542 g.

^d^ Mean, 272 g; median, 72 g; 99th percentile, 2136 g.

^e^ Mean, 245 g; median, 88 g; 99th percentile, 3573 g.

Increases in opioid overdose deaths were associated with increases in fentanyl or carfentanil seizures from 2014 until mid-2017 (Figure). Heroin seizures not containing fentanyl or carfentanil decreased consistently from 2014 to 2017, whereas heroin seizures containing fentanyl or carfentanil increased steadily. The adjusted multivariate, generalized, autoregressive, conditional-heteroskedasticity model shows that fentanyl seizures were significantly associated with overdose deaths, with every additional fentanyl seizure associated with an increase in deaths, with a continuous coefficient of 0.58 (95% CI, 0.41-0.74; *P* < .001). Carfentanil seizures were the only other significant covariate, with a coefficient of 0.34 (95% CI, 0.12-0.51; *P* = .002).

**Figure.**
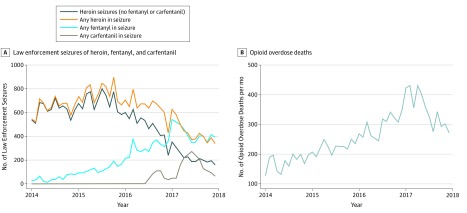
Opioid Overdose Deaths and Law Enforcement Seizures of Heroin, Fentanyl, and Carfentanil, Ohio, 2014 to 2017 A, Law enforcement seizures of heroin, fentanyl, and carfentanil are shown by month. B, Opioid overdose deaths are shown by month.

## Discussion

By integrating overdose mortality data from Ohio’s Vital Statistics System with state crime laboratory data from Ohio’s Bureau of Criminal Investigation, we demonstrate a significant association between law enforcement drug seizures and overdose deaths in Ohio from 2014 to 2017. To our knowledge, this is the first study to offer an empirical basis for using crime laboratory data as a viable indicator of opioid overdose deaths. This analysis is limited by the administrative character of crime laboratory data, which, like other institutional data systems (eg, electronic health records), are subject to internal measurement error. Because quantitative testing was not performed by Ohio’s Bureau of Criminal Investigation, the analysis could not identify percentage amounts of fentanyl and carfentanil in heroin, cocaine, and methamphetamine seizures and, thus, was unable to determine whether fentanyl adulteration was intentional or the result of unintentional trace contamination. The absence of drug seizures from private laboratories limits a comprehensive portrait across Ohio; however, the strength of our findings and large sample size suggest that our conclusions would not change materially by including samples from private laboratories. Our analyses were limited to Ohio and may not generalize beyond this state.

These findings underscore the importance of partnerships between public health and public safety to address the opioid overdose epidemic. Active data sharing between law enforcement and public health agencies can facilitate timely, actionable data to identify fentanyl hot spots and coordinate rapid responses that could limit overdose mortality.

## Notes

Supplement. **eAppendix.** Model Specification and Results
