# Error in Author’s Academic Degree

**DOI:** 10.1001/jamanetworkopen.2019.19971

**Published:** 2019-12-27

In the Research Letter titled “Association of Law Enforcement Seizures of Heroin, Fentanyl, and Carfentanil With Opioid Overdose Deaths in Ohio, 2014-2017,”^1^ published November 8, 2019, an incorrect academic degree was listed in the byline for Jolene DeFiore-Hyrmer. Ms DeFiore-Hyrmer’s degree should have been listed as MPH. This article has been corrected.^1^
