# Supplementary material for: Association of Law Enforcement Seizures of Heroin, Fentanyl, and Carfentanil With Opioid Overdose Deaths in Ohio, 2014-2017
Source: JAMA Netw Open. 2019 Nov 8;2(11):e1914666. doi: 10.1001/jamanetworkopen.2019.14666 (PMC6902770; doi:10.1001/jamanetworkopen.2019.14666)
Supplement: Supplement. — eAppendix. Model Specification and Results [file jamanetwopen-2-e1914666-s001.pdf]

Supplementary Online Content

Zibbell JE, Aldridge AP, Cauchon D, DeFiore-Hyrmer J, Conway KP. Association of law enforcement seizures of heroin, fentanyl, and carfentanil with opioid overdose deaths in Ohio, 2014-2017. *JAMA Netw Open*. 2019;2(11):e1914666. doi:10.1001/jamanetworkopen.2019.14666

eAppendix. Model Specification and Results

This supplementary material has been provided by the authors to give readers additional information about their work.

© 2019 Zibbell JE et al. *JAMA Network Open.*

eAppendix: Model Specification and Results

MGARCH is multivariate generalized autoregressive conditional heteroskedasticity used to model autocorrelation in time series analysis. The unit of analysis is a single month in Ohio covering four years. We estimated multiple models showing the inclusion of additional covariates. Our final model adds a one-month lag of fatal opioid overdose deaths. The main covariates of interest are *the number of seizures with heroin present but no fentanyl or carfentanil present*, the number of *seizures with fentanyl*, and the number with *carfentanil*. In building to our final specification, we added linear and quadratic time trends, the number of seizures with cocaine, the number with methamphetamine, and the number of all other exhibits excluding opioids, cocaine, and methamphetamine. Finally, we included monthly measures of prescribed opioids from the Ohio Automated Rx Reporting System (OARRS), which is the state's prescription drug monitoring program, to control for availability of prescription opioids (i.e., the legal opioid supply) that could have contributed to the opioid overdose death rate. The data were provided by OARRS and included the medication details needed to convert all opioids into Morphine Milligram Equivalent (MME) dosages. Two MME measures were used for the model covariates: the total MMEs per month for all opioids save fentanyl and MME amounts of fentanyl.

© 2019 Zibbell JE et al. *JAMA Network Open.*
